# Treatment of schizophrenia evaluated via the pharmacopsychometric triangle—An integrative approach with emphasis on well-being and functioning

**DOI:** 10.1038/s41537-023-00420-6

**Published:** 2023-12-16

**Authors:** Pernille Kølbæk, Ole Mors, Christoph U. Correll, Søren D. Østergaard

**Affiliations:** 1 Psychosis Research Unit, Aarhus University Hospital—Psychiatry, Palle Juul-Jensens Boulevard 175, 8200 Aarhus, Denmark; 2 Department of Clinical Medicine, Aarhus University, Palle Juul-Jensens Boulevard 82, 8200 Aarhus N, Denmark; 3 Division of Psychiatry Research, The Zucker Hillside Hospital, 75–59 263rd Street, Glen Oaks, NY 11004 USA; 4 Department of Psychiatry and Molecular Medicine, Donald and Barbara Zucker School of Medicine at Hofstra/Northwell, 500 Hofstra Blvd, Hempstead, NY 11549 USA; 5 Department of Child and Adolescent Psychiatry and Psychotherapy, Charite Universitätsmedizin, Augustenburger Platz 1, 13353 Berlin, Germany; 6 Department of Affective Disorders, Aarhus University Hospital—Psychiatry, Palle Juul-Jensens Boulevard 175, 8200 Aarhus, Denmark

**Keywords:** Schizophrenia, Psychosis

## Abstract

Quantification of treatment response is crucial to optimize outcomes for patients with schizophrenia. In this study, we evaluated the relationship between quantitative measures of clinician-rated symptom severity and self-rated side effects, well-being, and functioning among inpatients with schizophrenia using the six-item version of the Positive and Negative Syndrome Scale (PANSS-6), the Glasgow Antipsychotic Side-effect Scale (GASS), the WHO-Five Well-being Index (WHO-5), and the Sheehan Disability Scale (SDS). All measurements were conducted as close to admission and discharge as possible. Well-being and functioning were found to be most strongly associated with the additive effect of symptoms and side effects, while changes in side effects, well-being, and functioning appeared to be relatively independent from changes in symptom severity. The use of both symptom and side effect measures should inform clinical decision-making in the treatment of schizophrenia, as it has the potential to optimize functioning and well-being.

## Introduction

Schizophrenia is a severe mental disorder characterized by symptoms such as delusions, hallucinations, disorganized thinking, blunted affect, and social withdrawal^1^. Antipsychotics represent a cornerstone in the treatment of schizophrenia^2^. Unfortunately, both residual symptoms of the condition itself and potential side effects of antipsychotics are associated with reduced functioning and quality of life^3–6^. Rating scales are used to quantify symptom severity and other treatment outcomes in clinical trials for schizophrenia. However, the use of rating scales in routine clinical practice has yet to gain momentum. Possible reasons include a lack of psychometrically valid and clinically relevant clinician-rated and self-rated scales, time constraints, and a lack of formal training among clinical staff^7–9^. This situation impedes the translation of research into clinical practice^10^, and efforts to rectify it were among the forces driving the conceptualization of measurement-based care (MBC)^11,12^, i.e., the systematic administration of rating scales and the subsequent use of these ratings to guide individualized clinical decision making^12–14^. MBC has primarily been studied in relation to affective disorders, where the results have been promising^12,15,16^. Most notably perhaps, as demonstrated by the randomized controlled trial by Guo et al.^17^. showing the superiority of MBC over treatment-as-usual in achieving response and remission among outpatients with major depression.

While MBC in psychiatry has traditionally and predominantly focused on assessment of symptom severity, the treatments employed affect a range of other domains that are crucially important to quantify and monitor—and thereby optimize—successful treatment outcomes. In 2009, Bech suggested the so-called pharmacopsychometric triangle as a way of applying psychometrics to clinical psychiatry^18^. This model proposes use of rating scales to measure (i) the desired effects of psychotropic drugs—i.e., reduction in the severity of the core symptoms of the disorder, (ii) undesirable drug effects (side effects), and (iii) health-related quality of life assessments, such as self-perceived psychological well-being. In turn, the model uses these measures to inform clinical decision-making with the ultimate purpose of improving the quality of life. The pharmacopsychometric triangle has proven valuable in the treatment of depression by providing an integrated overview of treatment effects^12,19^. Specifically, Bech and colleagues employed the pharmacopsychometric triangle to assess the effectiveness of different augmentation therapies for depression. This includes the comparison of bupropion sustained release (SR) and buspirone augmentation of citalopram, finding that bupropion-SR was superior in terms of antidepressive efficacy, side effects, and quality of life^20^. Likewise, a study evaluating the effect of transcranial pulsed electromagnetic fields (T-PEMF) concomitant with antidepressants, demonstrated that active T-PEMF was superior to sham within the pharmacopsychometric triangle, with a clinically significant effect size of −0.48 on the 5-item World Health Organization Well-being Index (WHO-5) (higher scores reflecting higher quality of life, explaining the negative effect size), and 0.91 and 0.90 on the clinician-rated 17-item Hamilton depression scale and the self-reported 6-item Hamilton depression scale, respectively^19^.

Balancing the desired and undesired effects of pharmacological treatment remains a significant challenge in treating schizophrenia^1,3,4^. Recent research by Baandrup et al. has identified and classified subjective and objective outcome measures within the pharmacopsychometric triangle^21^, while citing the importance of including measures for medication side effects in addition to measures for symptom burden and quality of life. To our knowledge, only one prior study has evaluated treatment of schizophrenia in the context of the pharmacopsychometric triangle. However, this study was based on re-analysis of data from a placebo-controlled clinical trial in which the quality of life domain was assessed only via a proxy measure, i.e., a clinician-rated symptom subscale^22^. Moreover, associations between symptoms and quality of life and between symptoms and side effects were not assessed separately for positive and negative symptoms.

### Aims of the study

This study aimed to apply the pharmacopsychometric triangle to assess the relationship and potential covariation among the following: (i) core symptom severity assessed via the clinician-rated six-item Positive And Negative Syndrome Scale (PANSS-6)^23–25^, including its positive and negative symptom subscales, (ii) self-reported side effects via the Glasgow Antipsychotic Side-effect Scale (GASS), and (iii), self-reported quality of life via the WHO five-item well-being index (WHO-5) and the Sheehan Disability Scale (SDS). The study sample comprised inpatients with schizophrenia receiving standard care at a Danish psychiatric university hospital.

## Results

A total of 77 inpatients with schizophrenia participated in the study. Of these, 63 individuals (82%) completed the self-reported questionnaires at least once (yielding a total of 92 completed questionnaires, i.e., 57 at baseline close to admission, and 35 at endpoint close to discharge). The mean age of the 63 respondents was 35.5 years (standard deviation(SD) = 11.4), 56% were male (*n* = 35), and 68% (*n* = 43) had a diagnosis of paranoid schizophrenia. The vast majority of the respondents, i.e., 98% (*n* = 62), were treated with psychotropic medications (97% with antipsychotic medications), while 92% (*n* = 58) were treated with a second-generation antipsychotic, 21% (*n* = 13) were treated with a first-generation antipsychotic, and 46% (*n* = 29) were treated with an antidepressant in addition to an antipsychotic. The respondents (*n* = 63) and non-respondents (*n* = 14) did not differ significantly with regard to age (35.4 vs. 33.2 years, *p* = 0.518), sex (*p* = 0.550), PANSS-6 total scores (18.8 vs. 18.8, *p* = 0.986), or GAF-F scores (47 vs. 46, *n* = 45, *p* = 0.973).

Table 1 lists the baseline means of the PANSS-6 total and subscale scores, GAF-S and GAF-F scores, SDS total and subscale scores, WHO-5 total score, and GASS total score. Stratification on sex and age (<32 and ≥32 years [median split]) revealed no difference between males and females or between those aged <32 or ≥32 years, respectively. At baseline, the mean GAF-F score was 46.6 (SD = 15.3) (*n* = 45), indicating a severe impairment in functioning. The mean WHO-5 total score at baseline was 40.4 (SD = 21.9) (*n* = 57), and 67% of respondents (*n* = 38) had a WHO-5 total score <50 (poor well-being). Among those with a WHO-5 score <50, 55% (*n* = 21) were treated with an antidepressant.

**Table 1:** Baseline scores and baseline-endpoint differences on the PANSS-6, GAF, SDS, WHO-5 and GASS (mean ± SD).

Baseline (total group)					Scale range	Interpretation of high score	Baseline-endpoint change (follow-up group)			
	Total (*n* = 44–77)	Male (*n* = 28–44)	Female (*n* = −16–33)	*p*-value				Diff	95%CI	*p*-value
PANSS-6 total (*n* = **77)**	18.8 ± 4.6	18.6 ± 4.7	19.0 ± 4.4	0.711	6–42	Bad	PANSS-6 total (*n* = 50)	−0.86	[−2.01;0.29]	0.138
Positive subscale	10.6 ± 3.4	10.3 ± 3.6	11.0 ± 3.1	0.373	3–21	Bad	Positive subscale	−0.74	[−1.65;0.17]	0.108
Negative subscale	8.2 ± 3.0	8.4 ± 3.3	8.1 ± 2.5	0.661	3–21	Bad	Negative subscale	−0.12	[−0.84;0.60]	0.740
GAF-S (*n* = 44)	38.7 ± 14.2	40.4 ± 14.7	35.8 ± 13.4	0.300	1–100	Good	GAF-S (*n* = 13)	4.61	[−4.64;13.88]	0.230
GAF-F (*n* = 45)	46.6 ± 15.3	47.2 ± 15.0	45.6 ± 16.3	0.749	1–100	Good	GAF-F (*n* = 13)	5.15	[−2.50;12.80]	0.168
SDS total (*n* = 56)	16.6 ± 7.7	15.8 ± 7.9	17.5 ± 7.5	0.426	0–30	Bad	SDS total (*n* = 28)	−2.39	[−5.95;1.16]	0.179
SDS work	5.8 ± 3.1	5.3 ± 3.2	6.3 ± 3.0	0.269	0–10	Bad	SDS work	−0.79	[-2.22;0.65]	0.273
SDS social	5.8 ± 2.9	5.7 ± 2.9	5.9 ± 3.0	0.777	0–10	Bad	SDS social	−0.89	[−2.19;0.40]	0.168
SDS family	5.0 ± 2.8	4.8 ± 2.6	5.3 ± 3.0	0.500	0–10	Bad	SDS family	−0.71	[−2.00;0.57]	0.263
WHO-5 (*n* = **57)**	40.3 ± 22.0	42.6 ± 19.6	37.5 ± 24.5	0.392	0–100	Good	WHO-5 (*n* = **35)**	11.44	[4.04;18.85]	**0.004**
GASS (*n* = 54)	20.3 ± 9.3	20.6 ± 10.1	20.0 ± 8.3	0.781	0–66	Bad	GASS (*n* = 26)	−2.46	[−5.61;0.69]	0.120

*PANSS-6* Six-item positive and negative syndrome scale, *GAF* Global assessment of functioning scale, *SDS* Sheehan’s disability scale, *WHO-5* Five-item World Health Organization well-being index, *GASS* Glasgow antipsychotic side-effect Scale.

*P*-values < 0.05 are in bold text.

The Spearman’s correlation coefficient (rho) for the comparison of GAF-S and PANSS-6 total scores at baseline (*n* = 44) was −0.36, *p* = 0.016. The association was strongest between the GAF-S and the positive subscale of PANSS-6 (rho = −0.43, *p* = 0.003) and weaker between the GAF-S and the negative subscale of PANSS-6 (rho = −0.12, *p* = 0.456). The correlation between GAF-F and SDS was weak and not statistically significant (rho = 0.08, *p* = 0.644). The correlation also failed to reach statistical significance between any of the three SDS subscales and for GAF-F.

**Fig. 1:**
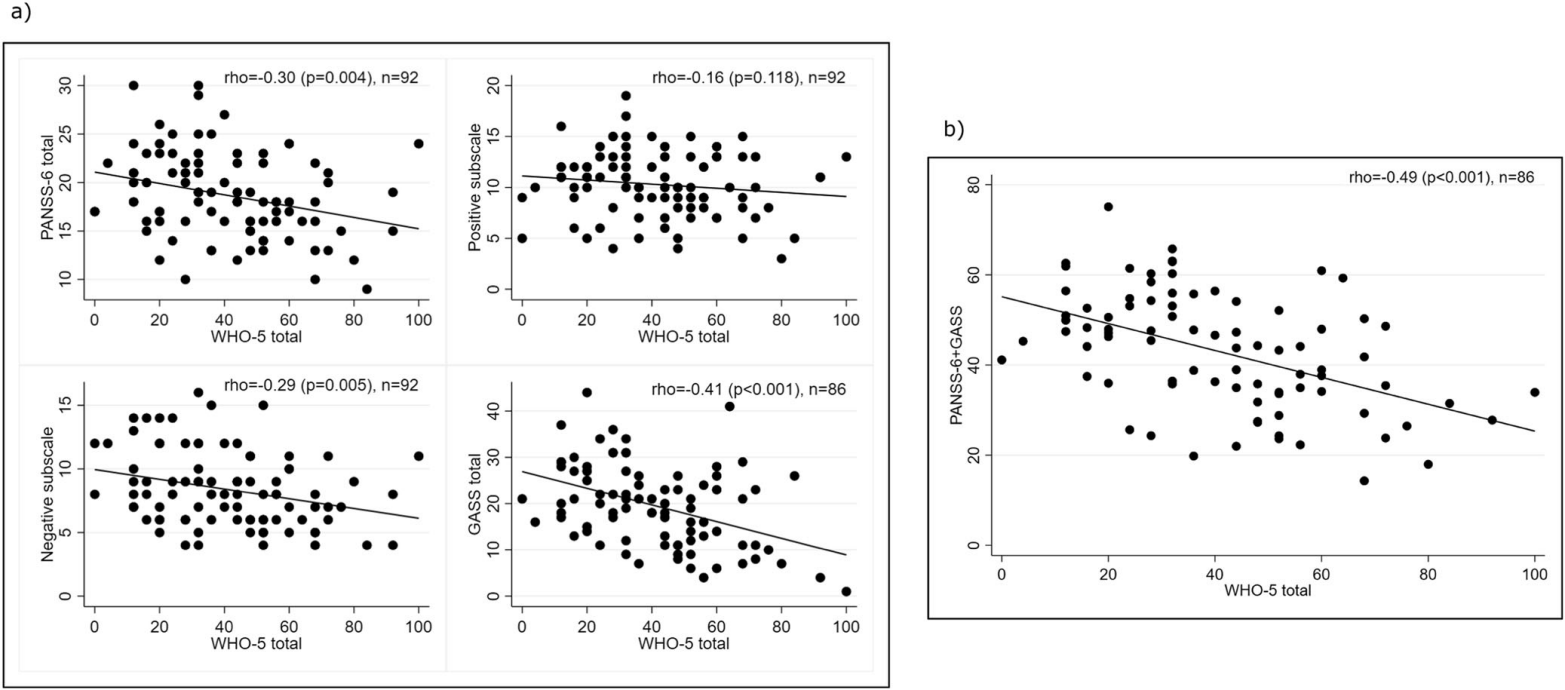
The association between core schizophrenia symptoms, side effects, and quality of life. Spearman’s rank correlation coefficient (rho) for (**a**) the PANSS-6 total and subscale scores and the GASS total scores versus the WHO-5 total score and (**b**) the additive effect of PANSS-6 + GASS versus the WHO-5 total score.

Figure 1 shows the correlations between the PANSS-6 total score, its positive and negative subscales scores, the GASS, and the WHO-5. Among these correlations, the additive effect of PANSS-6 + GASS vs. WHO-5 demonstrated the strongest association (rho = −0.49, *p* < 0.001, *n* = 86). The Spearman’s rank correlation coefficients for the relationship between the individual PANSS-6 items and the WHO-5 total scores at baseline (*n* = 57) were as follows: Item P1 Delusions: rho = −0.25, *p* = 0.06; Item P2 Conceptual disorganization: rho = 0.29, *p* = 0.03; Item P3 Hallucinatory behavior: rho = −0.36, *p* = 0.005; Item N1 Blunted affect: rho = −0.34, *p* = 0.001; Item N4 Passive/apathetic social withdrawal: rho = −0.18, *p* = 0.18; and Item N6 Lack of spontaneity and flow of conversation = −0.16, *p* = 0.24.

**Fig. 2:**
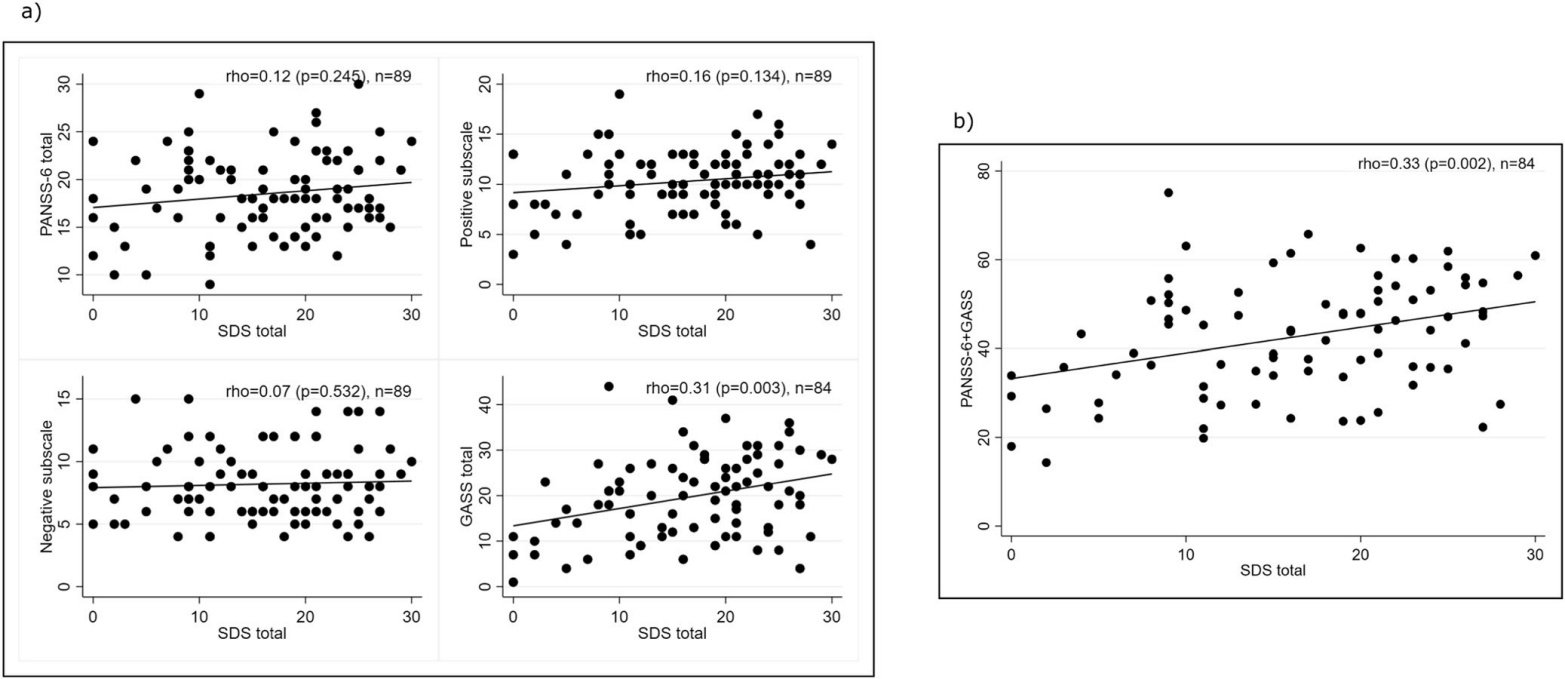
The association between core schizophrenia symptoms, side effects, and functioning. Spearman’s rank correlation coefficient (rho) for (**a**) the PANSS-6 total and subscale scores and the GASS total scores versus the SDS total score and (**b**) the additive effect of PANSS-6 + GASS versus the SDS total score.

Figure 2 presents the correlations between the PANSS-6 total score, its positive and negative subscale scores, GASS scores, and SDS scores. Among those variables, the additive effect of PANSS-6 + GASS vs. SDS demonstrated the strongest association (rho = 0.33, *p* = 0.002, *n* = 84).

The Spearman’s rank correlation coefficients for the PANSS-6 total and subscale scores and the GASS total score were as follows: PANSS-6 total vs. GASS: rho = 0.13, *p* = 0.216; PANSS-6 positive subscale vs. GASS: rho = 0.35, *p* < 0.001; and PANSS-6 negative subscale vs. GASS: rho-0.21, *p* = 0.054. None of the Spearman’s rank correlation coefficients for baseline-endpoint differences were statistically significant. Supplementary material Table 1 lists all comparisons and 95% CI.

The WHO-5 and SDS total scores and the baseline-endpoint differences were moderately correlated (rho = −0.41, *p* < 0.001, *n* = 89 and rho = −0.47, *p* = 0.011, *n* = 28, respectively).

**Fig. 3:**
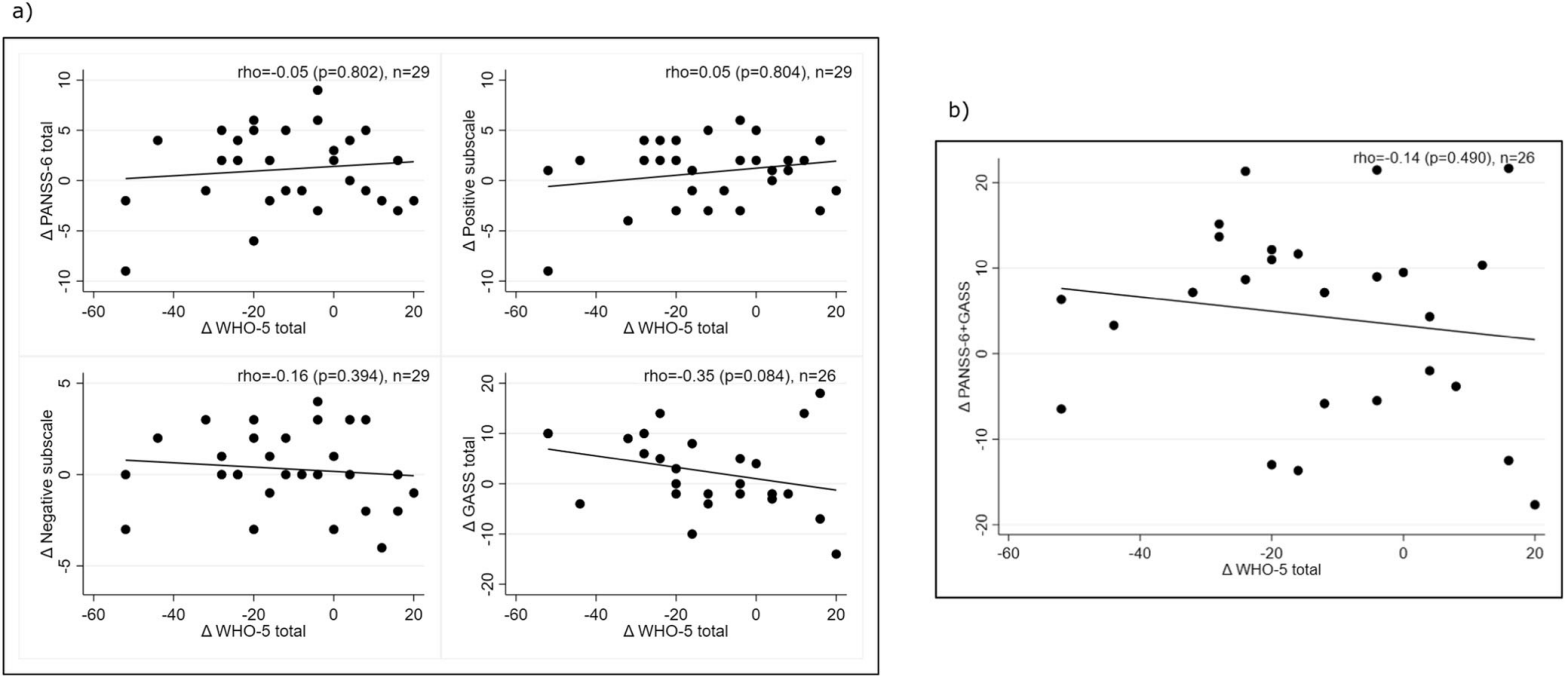
The association between changes in core schizophrenia symptoms, side effects, and quality of life. The Spearman’s rank correlation coefficient (rho) for the baseline-endpoint differences (Δ) on (**a**) the PANSS-6 total, the positive and negative subscale, and the GASS total scores versus the WHO-5 total score and (**b**) the additive effect of PANSS-6 + GASS versus the WHO-5 total score.

**Fig. 4:**
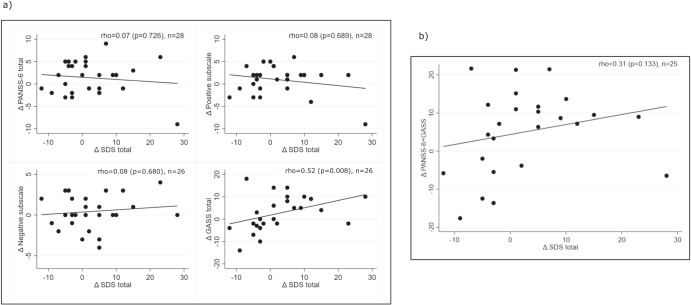
The association between changes in core schizophrenia symptoms, side effects, and functioning. The Spearman’s rank correlation coefficient (rho) for the baseline-endpoint differences (Δ) on (**a**) the PANSS-6 total, the positive and negative subscale, and the GASS total scores versus the SDS total score and (**b**) the additive effect of PANSS-6 + GASS versus the SDS total score.

The mean baseline-to-endpoint difference on the self-reported measures was 11.4 (SD = 19.5) for the WHO-5, −2.5 (SD = 7.8) for the GASS, and 2.4 (SD = 9.2) for the SDS. The 29 participants who completed the WHO-5 at both time points improved significantly (33.7 (SD = 17.0) at baseline vs. 45.1 (SD = 21.2) at endpoint, *p* = 0.004). Figures 3 and 4 illustrate the correlation between changes in baseline-to-endpoint scores on the PANSS-6, its subscales and GASS and the WHO-5, and SDS, respectively. The strongest correlation was found for the baseline-to-endpoint difference between GASS and SDS: rho = 0.52 (*p* = 0.008, *n* = 28). For the baseline-to-endpoint differences on the individual PANSS-6 items, a trend was only detected in the inverse relationship between the severity of N1 Blunted affect and well-being (WHO-5 total score, rho = −0.33, *p* = 0.087, [*n* = 29]).

## Discussion

The overall results of this study attest to the disability and diminished well-being associated with schizophrenia. The specific main findings were as follows: (i) psychological well-being and functioning were most strongly associated with the additive effect of core schizophrenia symptoms and antipsychotic side effects, (ii) changes in symptom severity alone were not correlated at the level of statistical significance with functioning, well-being, or side effects, (iii) levels of self-reported side effects and well-being were correlated at the level of statistical significance, and (iv) levels of self-reported side effects and functioning were correlated at the level of statistical significance, as were the changes between these measures over time.

Traditionally, measures of symptom severity have constituted the primary outcome in the evaluation of mental health treatment. Our results lend support to the importance of including additional outcomes when monitoring schizophrenia and its treatment effects. Notably, the additive effect of symptoms and side effects exhibited the strongest correlation with well-being, thereby supporting the key principle of the pharmacopsychometric triangle^18^, which identifies core symptom severity, side effects, and psychological well-being as its three cornerstones. Unlike studies of other mental disorders, such as mood disorders^26^, however, we found a less straightforward relationship between severity of symptoms and psychological well-being in schizophrenia; i.e., well-being did not necessarily increase as core symptom severity decreased and vice versa. Clinical explanations of this finding may include the presence of grandiose delusions, which may increase the perception of well-being, or lack of insight, which may, in some cases, prevent the affected individual from realizing the significant impact and serious consequence of the disease. Nevertheless, as reported previously^27^, the change in core symptom severity in our study population was minimal. Hence, the apparent independence of symptom severity and well-being may derive simply from the lack of variance in symptom severity over time. In addition, a relatively large portion of the study population (46%) was treated with antidepressant medication. Therefore, the overall improvement in their mental well-being may have been caused by an overall reduction in their symptoms of depression^9^. This possibility is supported by the multivariate analysis of a pooled sample comprising 886 individuals with schizophrenia by Priebe et al.^28^, who report statistically significant associations only between changes in depression/anxiety and hostility subscales and changes in quality of life measures.

The additive effect of symptoms and side effects also exhibited the strongest correlation with functioning, thereby underlining the importance of the integrated view of treatment effects, which the pharmacopsychometric triangle provides^18^. Conversely, the relationship between severity of core schizophrenia symptoms and functioning was not statistically significant, which aligns with the literature on this topic^26,29,30^. Interestingly, prior studies have suggested that anxiety and depressive symptoms are associated with the level of functioning among individuals with schizophrenia^31–33^ with the most pronounced impairments seen for the domains of work and social life^31^. These domains were also those who appeared to be most negatively affected among the participants of the present study. Taken together, this result underlines the importance of identifying and treating comorbid anxiety and depressive symptoms, as these comorbidities may represent an additional burden for individuals with an already compromised quality of life due to psychosis. Notably, although the participants of the present study improved significantly on the WHO-5 well-being index, their mean value at the endpoint assessment (42) was still considerably lower than the mean score for the general population of Denmark (62–70), but similar to other populations with schizophrenia^34–38^. These findings underscore the importance of self-reported quality of life as an independent outcome in the evaluation of treatment effect in schizophrenia, while also underlining the importance of monitoring the severity of concurrent depressive symptoms. Notably, for the measurement of depressive symptoms among individuals with schizophrenia, scales with the ability to discriminate depressive symptoms from negative symptoms, such as the Calgary Depression Scale for Schizophrenia^39^, are recommended.

Interestingly, the self-reported SDS score did not correlate with the clinician-rated GAF-F score. Notably in this regard, we did not test the reliability of staff conducting the GAF-F ratings, and studies have reported that the reliability of GAF-F rating is often not satisfactory in routine clinical settings^40^. However, an alternative explanation may be that the two measures capture different sub-constructs of functioning. Specifically, self-reported measures may predominantly measure the patient’s subjective experience of social dysfunction (e.g., loneliness and perceived social support), whereas clinician-rated scales may gauge more objective aspects of social dysfunction (e.g., social network size and social cognition assessed with tasks)^41,42^. Thus, both clinician-rated and patient-reported measures are clinically relevant since they may capture different but equally important constructs.

Including self-reported side effects in assessing the pharmacological treatment of schizophrenia is important because physicians may overestimate the tolerability of antipsychotic medication^37^. In the assessment of the relationship between clinician-rated symptoms and self-reported side effects, we found that positive symptoms were associated with side effects. Since positive symptoms are treatment responsive, an association between side effects and positive symptoms may reflect higher doses of antipsychotic medication. Conversely, the association between positive symptoms and side effects may reflect a lack of insight among these patients^43^, which could hinder their ability to differentiate between illness symptoms and side effects. Notably, the GASS has only been validated against the clinician-rated UKU in an outpatient sample^44^. Thus, future studies should assess the degree to which side effects reported by severely ill inpatients are, in fact, side effects and not symptoms of the illness being treated. While the additive effect of symptom severity and side effect burden on quality of life could provide valuable insights into the distinctions between antipsychotics and potentially lead to measurement-based care approaches in the treatment of schizophrenia, it is crucial to evaluate symptom severity and side effects separately to inform clinical decision-making^45^. For instance, in managing common side effects of antipsychotics, like dystonia and parkinsonism, strategies may include switching to an alternative medication to enhance treatment outcomes, with the ultimate goal of improving patients’ quality of life^46^. Although, psychological well-being and functioning were most strongly associated with the additive effect of core schizophrenia symptoms and antipsychotic side effects, the change over time was most strongly associated with change in antipsychotic side effect burden. While these results should be interpreted with caution due to small sample sizes, minimal change in core schizophrenia symptom severity, and short follow-up periods between assessments, they support a practice in which core schizophrenia symptoms and antipsychotic side effects are evaluated separately.

Self-reported side effects were also associated with well-being and functioning. Side effects of common antipsychotics include adverse mood effects, which may be captured by the WHO-5 as poor well-being. Conversely, the severity of depressive symptoms may also affect subjective evaluations of the severity of side effects^47^. Regardless, the association between side effects and well-being both in this study and in a previous study among outpatients with schizophrenia^44^ underlines the importance of granting equal weight to side effect and symptom evaluations. This is further emphasized by studies identifying side effects as one of the main reasons for non-adherence and treatment discontinuation^48^.

A number of limitations should be taken into account when interpreting the results of this study. First, it is important to note that this paper is based on secondary analyses of data from a study, which was not specifically designed to evaluate the treatment of schizophrenia from the perspective of the pharmacopsychometric triangle. Most notably in this regard, the absence of a specific intervention has likely contributed to the minimal change in core symptom severity from baseline to endpoint. Moreover, although the method of combining the scales is commonly used^49–51^ and feasible for clinical practice due to its simplicity (rescaling the scales and adding the scores), it may not be the ideal way to handle this. Hence, future studies should evaluate the impact of alternative strategies such as that suggested by Kraemer et al.^45^. Second, the sample size for the analysis of change over time was small (*n* = 29) although sufficient to conclude that scores on the GASS and SDS covary over time. The small sample size, differences in the reference periods between measures, and the minimal change on the PANSS-6 between the two points of assessment may collectively explain the lack of statistically significant associations between the PANSS-6 and the GASS, SDS and WHO-5. Also, it is important to note that the analysis comparing respondents and non-respondents may not fully capture potential heterogeneity within the non-respondent group since this group may comprise subpopulations with varying symptom profiles. Third, while this study exclusively incorporated a measure for self-reported side effects related to antipsychotics, it is equally important to assess side effects of other medications, such as antidepressants. To address this concern, we have developed the Aarhus Side Effect Assessment Questionnaire (ASAQ), a self-reported tool with global coverage across side effect domains and psychotropic drug classes and with strong measurement properties^52^. We intend to employ this tool in future studies and clinical practice. Self-report measures may hold several advantages over clinician-rated measures such as being independent of the availability of trained, reliable raters, being less time-consuming, and allowing for out-of-hospital assessments; however, self-report may be biased by participants’ lack of illness insight, which may challenge patients in differentiating between constructs under evaluation such as symptoms and medication-induced side effects. To reduce the impact of this bias, we employed the validated GASS, which has demonstrated high sensitivity and specificity among individuals with psychotic disorders using the clinician-rated UKU as a gold standard. Relatedly, there may be conceptual overlap between the measures of well-being and functioning. However, the SDS^53^ and the WHO-5^34^ have been validated in populations similar to the present study and hence, we find it reasonable to assume that these instruments measure the constructs of interest namely different subdomains of quality of life. Nonetheless, to gain clarity about the relationship between symptoms, side effects and quality of life, we evaluated these relationships separately for each of the subdomains of quality of life i.e., functioning and well-being. Fourth, the lack of assessment of affective and cognitive symptoms precluded this study from assessing their potential impact on well-being and functioning. Fifth, the diagnoses of the participants were provided by the treating psychiatrist and not confirmed by diagnostic interviews. However, diagnostic semi-structured interviews such as the Present State Examination and Schedules for Clinical Assessment in Neuropsychiatry^54^ are routinely used in clinical practice in Denmark. Moreover, a previous study has reported high validity of schizophrenia diagnoses in Danish medical records^55^. Sixth, excluding individuals under the influence of psychoactive substances including alcohol or being treated via involuntary measures at the time of potential recruitment and interview may reduce the generalizability of the results. The rationale behind applying these exclusion criteria was mainly for ethical reasons, i.e., to ensure that the consent to participate in the study was both informed and voluntary. Seventh and lastly, the sample included only Danish inpatients with schizophrenia. Therefore, the results of this study should be replicated in other settings, e.g., in other countries and among individuals with other psychotic disorders, and in other stages of illness.

In conclusion, the results of this study provide evidence for a relationship between self-reported side effects, functioning, and mental well-being among inpatients with schizophrenia. Somewhat counter-intuitively, neither of these measures was clearly associated with positive and negative symptom severity. However, the additive effect of symptom severity and side effects was more strongly associated with well-being than were side effects alone. Thus, the integrated overview of desired and undesirable treatment effects—as conceptualized by the pharmacopsychometric triangle—may aid decision making when treating individuals with schizophrenia in clinical practice.

## Methods

### Data source

Data for this analysis derive from a recent study, which aimed to validate ratings on the six-item version of the Positive And Negative Symptom Scale (PANSS-6) obtained via the Simplified Negative And Positive Symptoms Interview (SNAPSI)^27^. The data used for the present analysis are described briefly below and the sampling of participants is illustrated in Supplementary material Fig. 1.

### Participants

Participants were recruited from inpatient wards at the Department for Psychosis at Aarhus University Hospital—Psychiatry, Denmark from January 2018 to October 2019. Eligibility criteria were as follows: (i) meeting the International Classification of Disease, 10th Revision (ICD-10) criteria for schizophrenia according to the treating psychiatrist (verified by the diagnosis in the patient’s medical chart), (ii) being aged ≥ 18 years, and (iii) understanding written and spoken Danish. Patients were ineligible if they were (i) diagnosed with comorbid organic mental disorder (ICD-10: F0x.x), (ii) diagnosed with mental retardation (IQ < 70), (iii) influenced by psychoactive substances including alcohol at the time of potential recruitment (as per clinical assessment by the referring clinician), or (iv) being treated via involuntary measures at the time of potential recruitment.

All participants provided written informed consent. In Denmark, ethical review committee approval is not required for studies based on interviews and questionnaires. All data were processed and stored in accordance with the European Union General Data Protection Regulation.

### Measures

Participants were assessed as close to admission and discharge as possible. In the present study, the following measures were used:

#### The six-item positive and negative syndrome scale

The six-item Positive And Negative Syndrome Scale (PANSS-6) consists of the following items: P1 Delusion, P2 Conceptual disorganization, P3 Hallucinatory behavior, N1 Blunted affect, N4 Passive/apathetic social withdrawal, and N6 Lack of spontaneity and flow of conversation during a reference period of the preceding week^25^. In a recently published study, we found strong agreement between PANSS-6 ratings obtained via the SNAPSI and PANSS-6 ratings extracted from the full PANSS-30 ratings obtained via the comprehensive Structured Clinical Interview for PANSS^27^. Trained and reliable raters obtained the information for PANSS-6 ratings via the brief Simplified Positive And Negative Symptoms Interview (SNAPSI; ICC of the PANSS-6 total score was 0.74 [*F* = 2.84, *p* = 0.03])^10^. Detailed information regarding the raters (including training and inter-rater reliability) can be found elsewhere^23^. The information obtained from each SNAPSI patient interview was supplemented by the SNAPSI informant interview of a staff member.

#### The Glasgow antipsychotic side-effect scale

The Glasgow Antipsychotic Side-effect Scale (GASS) is a self-report questionnaire consisting of 22 questions probing for side effects to antipsychotic agents^56^. The frequency of each side effect is rated on a four-point Likert scale ranging from 0 (Never) to 3 (Every day). The reference period for 20 GASS items is the preceding week. For the remaining two items, “weight gain” and “menstrual disturbances,” the reference period is the preceding three months. The frequency rating is supplemented by an assessment of subjective distress associated with each reported side effect. Recently, we found that among outpatients with psychotic disorders, self-reported side effects using the GASS exhibited strong agreement with clinician-rated side effects assessed with the Udvalg for Kliniske Undersøgelser (UKU) side effect rating scale^44^.

#### The Sheehan disability scale

The Sheehan Disability Scale (SDS) is a three-item self-reported questionnaire assessing disability across three domains: work, social life, and family life^53^. The degree to which the patient’s symptoms have disrupted each domain over the time frame of interest is rated on a 11-point Likert scale (with visual-spatial, numeric, and verbal descriptive anchors ranging from 0 [Not at all] to 10 [Extremely]). The reference period for the Danish version of the SDS is the preceding two weeks. Previous studies on the SDS have reported good psychometric properties with high internal consistency, reliability and construct validity^57^.

#### The five-item World Health Organization well-being index

The five-item World Health Organization Well-being Index (WHO-5) is a self-reported questionnaire measuring psychological well-being^34,58^. The scale contains five statements (i) “I have felt cheerful and in good spirits,” (ii) “I have felt calm and relaxed,” (iii) “I have felt active and vigorous,”(iv) “I woke up feeling fresh and rested,” and (v) “My daily life has been filled with things that interest me.” The reference period is the preceding two weeks and each statement is rated on a six-point Likert scale ranging from a score of 0 (At no time) to a score of 5 (All of the time). The total score, obtained by multiplying the sum score (range: 0–25) by 4, yields a range of 0–100 with higher scores indicating greater psychological well-being. The WHO-5 has been validated against the World Health Organization Quality of Life assessment among individuals with psychotic disorders and has demonstrated satisfactory levels of reliability and validity^59^.

#### The Global assessment of functioning scale

The Global Assessment of Functioning Scale (GAF) is a clinician-rated scale of symptom severity and functioning. The reference period is the preceding week and functioning is rated on a single dimension going from 100 (extremely high functioning) to 1 (severely impaired)^60^. To isolate functioning from symptom severity, we used the version of GAF that includes separate scores for symptoms (GAF-S) and for functioning (GAF-S). After attending an instruction session on the principles of GAF rating (held prior to study initiation on the wards where study participants were recruited), inpatient staff members performed the GAF ratings.

### Statistical analysis

To test for differences between respondents (i.e., participants who completed the self-reported questionnaires at least once) and non-respondents, a two-sample t-test was employed to compare means of continuous variables (age, PANSS-6 total score, and GAF-F score), and Pearson’s chi-squared test was employed for categorical variables (sex). Baseline-endpoint differences in the respondent group at follow-up were tested via a paired t-test. The pairwise relationship between the following measures were analysed using Spearman’s rank correlation: PANSS-6 (total score, positive and negative subscale scores, and individual item scores), WHO-5 (total score), SDS (total score and subscale scores), and GASS (total score). Additionally, (i) the GAF-S and the PANSS-6 total and subscale scores and (ii) the GAF-F and the SDS scores were compared using Spearman’s rank correlation. 95% two-tailed confidence intervals were calculated using a bootstrap method. All analyses were repeated using the baseline-endpoint difference for each measure to test for potential covariation of the measures over time. To test for an additive effect of symptoms and side effects on psychological well-being, the sum of the PANSS-6 total score and the GASS total score (PANSS-6 + GASS) was compared to the WHO-5 total score and the SDS total score, respectively, using Spearman’s rank correlation. In order to weight symptoms and side effects equally in the combined PANSS-6 + GASS score, the PANSS-6 total score was rescaled from a range of 6–42 to a range of 0–66 (the range of the GASS) by subtracting 6 and multiplying by 1.83 (the ratio between the highest possible total scores of the GASS and PANSS-6).

### Supplementary information

Supplementary material

## Data Availability

The participants of this study did not agree to their data being shared publicly.
