# Supplementary material for: Treatment of schizophrenia evaluated via the pharmacopsychometric triangle—An integrative approach with emphasis on well-being and functioning
Source: Schizophrenia (Heidelb). 2023 Dec 16;9(1):88. doi: 10.1038/s41537-023-00420-6 (PMC10725501; doi:10.1038/s41537-023-00420-6)
Supplement: Supplementary file 1 — Supplementary material [file 41537_2023_420_MOESM1_ESM.pdf]

Supplementary material

Supplementary Figure 1. Sampling of participants.

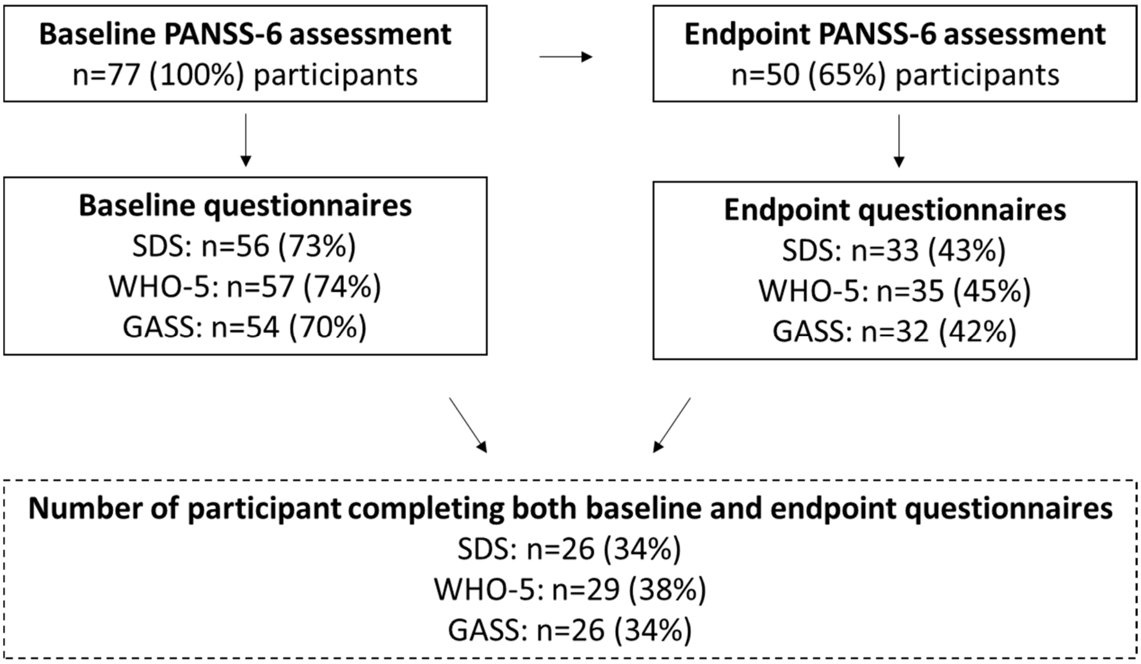

Abbreviations used: PANSS-6: Six-item Positive And Negative Syndrome Scale; SDS: Sheehan's Disability Scale; WHO-5: Five-item World Health Organization Well-being Index; GASS: Glasgow Antipsychotic Side-effect Scale.

Supplementary Table 1. Spearman's rank correlation coefficients (95% CIs).

		Total scores					
		PANSS-6 tot	PANSS-6 pos	PANSS-6 neg	GASS	SDS	WHO-5
Change in scores	PANSS-6 tot		**0.72 (0.63, 0.82)**	**0.62 (0.50, 0.74)**	0.13 (-0.07, 0.34)	0.12 (-0.09, 0.34)	-0.30 (-0.49, -0.11)
	PANSS-6 pos	**0.75 (0.62, 0.88)**		-0.03 (-0.21, 0.14)	0.35 (0.17, 0.53)	0.16 (-0.06, 0.38)	-0.16 (-0.36, 0.04)
	PANSS-6 neg	**0.66 (0.49, 0.83)**	0.03 (-0.26, 0.33)		-0.21 (-0.41, -0.01)	0.07 (-0.14, 0.28)	-0.29 (-0.48, -0.10)
	GASS	0.08 (-0.32, 0.47)	0.33 (-0.06, 0.71)	-0.15 (-0.57, 0.26)		0.31 (0.11, 0.52)	-0.41 (-0.60, -0.22)
	SDS	0.07 (-0.33, 0.47)	0.08 (-0.36, 0.51)	0.08 (-0.32, 0.48)	**0.52 (0.14, 0.90)**		-0.41 (-0.60, -0.21)
	WHO-5	-0.05 (-0.44, 0.34)	0.05 (-0.34, 0.43)	-0.16 (-0.55, 0.22)	-0.35 (-0.81, 0.11)	-0.47 (-0.82, -0.13)	

Abbreviations used: PANSS-6: Six-item Positive And Negative Syndrome Scale (tot: total score; pos: positive subscale score; neg: negative subscale score); SDS: Sheehan's Disability Scale; WHO-5: Five-item World Health Organization Well-being Index; GASS: Glasgow Antipsychotic Side-effect Scale.
